# Molecular Cloning, Biochemical Characterization, and Structural Insights into a Flavonoid-Associated Class II 4-Coumarate:CoA Ligase from *Sageretia thea*

**DOI:** 10.4014/jmb.2604.04014

**Published:** 2026-06-29

**Authors:** Yo-Heun Kim, Shin Ae Lee, Ji-Eun Kim, Minseok Cha, Jihoon Jo, Kyung Jun Lee, Soo-Jung Kim

**Affiliations:** 1 Department of Integrative Food, Bioscience and Biotechnology, Chonnam National University, Gwangju 61186, Republic of Korea; 2 Division of Biodiversity Conservation, Honam National Institute of Biological Resources, Jeollanam-do 58672, Republic of Korea; 3 Research Center for Biological Cybernetics, Chonnam National University, Gwangju 61186, Republic of Korea; 4 Division of Genetic Diversity, Honam National Institute of Biological Resources, Jeollanam-do 58672, Republic of Korea; 5 Division of Bioresource Integration Research, Honam National Institute of Biological Resources, Jeollanam-do 58672, Republic of Korea

**Keywords:** *Sageretia thea*, 4-Coumarate:CoA ligase, Phenylpropanoid pathway, Flavonoid biosynthesis, Metabolic engineering, Molecular docking

## Abstract

The enzymatic regulation of phenylpropanoid metabolism is a critical determinant of flavonoid biosynthesis in medicinal plants. *Sageretia thea* is valued for its pharmacological properties associated with flavonoid production; however, the molecular mechanisms governing pathway entry remain poorly understood. In this study, we identified and functionally characterized a novel 4-coumarate:CoA ligase (St4CL1) from *S. thea*. Phylogenetic analysis classified St4CL1 as a Class II isoform, a group typically associated with flavonoid biosynthesis, and multiple sequence alignment revealed the presence of highly conserved functional motifs, including the AMP-binding and catalytic domains. Recombinant St4CL1, heterologously expressed in *Escherichia coli*, exhibited a strong preference for Mg^2+^ and optimal catalytic activity at pH 7.0–8.0 and 35°C. Substrate specificity analysis revealed that St4CL1 exhibited the highest relative activity toward *p*-coumaric acid, supporting its role in directing carbon flux into the flavonoid biosynthetic pathway. Homology modeling and molecular docking revealed a conserved substrate-binding pocket within the inter-domain cleft, and *p*-coumaric acid was the substrate to form a salt-bridge contact at the carboxylate-binding site, providing a structural rationale consistent with its preferred turnover. Collectively, these findings provide the first molecular evidence of the phenylpropanoid entry step in *S. thea* and identify St4CL1 as a promising enzymatic target for metabolic engineering to enhance flavonoid production.

## Introduction

**Fig. 1:**
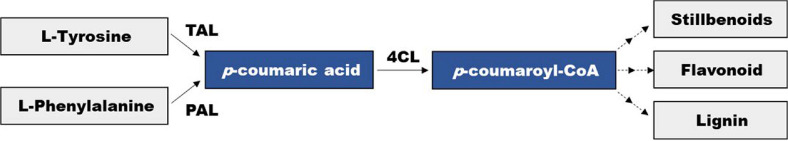
Enzymatic reaction of 4-coumarate:CoA ligase (4CL) generating *p*-coumaroyl-CoA, the branch-point intermediate of phenylpropanoid-derived pathways. L-Tyrosine and L-phenylalanine are converted to *p*-coumaric acid and subsequently activated to *p*-coumaroyl-CoA by 4CL, which serves as a central branch point for stilbene, flavonoid, and lignin biosynthesis. TAL, L-tyrosine ammonia-lyase; PAL, Phenylalanine ammonia lyase; 4CL, 4-coumarate:CoA ligase.

Secondary metabolism in plants produces a wide array of bioactive compounds that support survival by mediating defense against herbivores, protecting against ultraviolet radiation, and attracting pollinators [1]. Among these, the phenylpropanoid pathway is one of the most prominent metabolic routes, originating from phenylalanine synthesized via the shikimate pathway [2, 3]. This pathway generates precursors for diverse aromatic metabolites, ranging from structural polymers such as lignin, which provide mechanical support and vascular integrity, to water-soluble flavonoids and stilbenoids, which function as antioxidants and stress-response mediators (Fig. 1)[4]. In recent years, phenylpropanoid-derived compounds such as curcumin, resveratrol, and quercetin have attracted considerable attention in the pharmaceutical and nutraceutical industries due to their numerous therapeutic properties, including anti-inflammatory, anticancer, and antiviral activities [5]. Understanding the molecular mechanisms that regulate this pathway is therefore essential for metabolic engineering strategies aimed at enhancing the production of high-value metabolites.

**Fig. 2:**
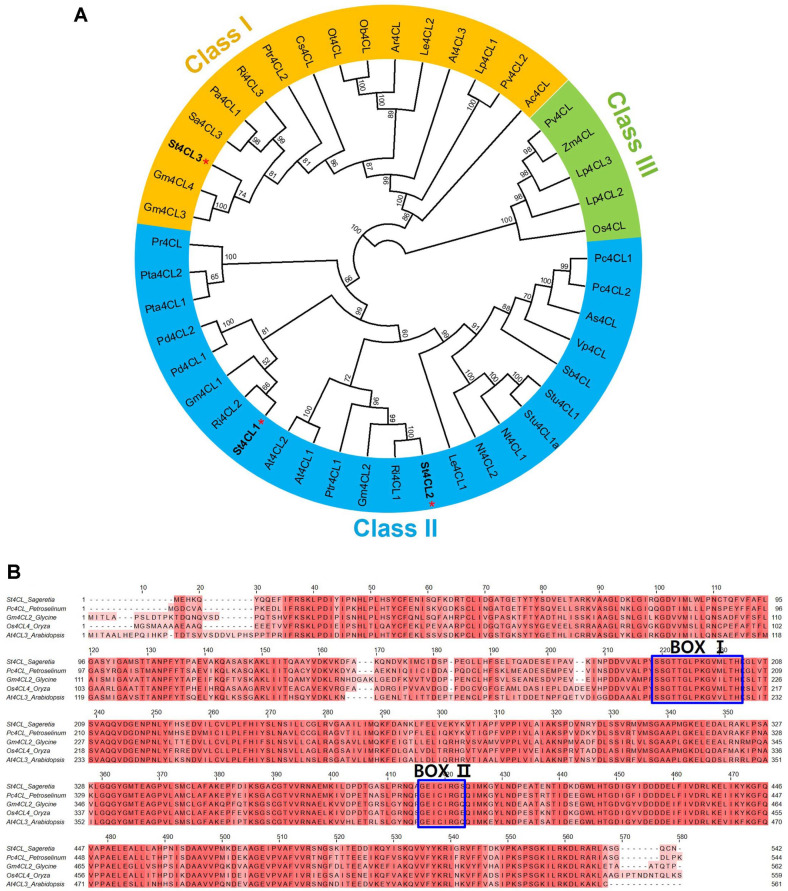
Phylogenetic analysis and multiple sequence alignment of St4CLs. (**A**) Phylogenetic tree of 4CL proteins from *S. thea* and other plant species. Three *S. thea* 4CLs are indicated with red asterisks. Numbers at the nodes show bootstrap support values. (**B**) Multiple sequence alignment of St4CL1 from *S. thea* with 4CL orthologs from *Arabidopsis thaliana*, *Glycine max*, *Oryza sativa*, and *Petroselinum crispum*. Blue boxes indicate conserved motifs (Box I and Box II). Identical residues are highlighted in red.

The enzyme 4-coumarate:CoA ligase (4CL; EC 6.2.1.12) represents a pivotal branch-point enzyme in the core phenylpropanoid pathway, generating activated thioesters [6]. Specifically, 4CL catalyzes the ATP-dependent activation of hydroxycinnamic acids—including *p*-coumaric acid, caffeic acid, and ferulic acid—into their corresponding CoA thioesters through a two-step reaction [7]. The phenylpropanoid pathway, initially catalyzed by phenylalanine ammonia-lyase [8], ultimately leads to this activation step by 4CL. This crucial step diverts carbon flow (via the shikimate-derived phenylalanine) into downstream branches of the phenylpropanoid pathway, leading to lignin and flavonoid biosynthesis [9]. In many plant species, 4CLs are encoded by multigene families. Isoforms within these families often display distinct substrate specificities and expression patterns, thereby channeling intermediates into different branches of the phenylpropanoid pathway [10]. Phylogenetically, 4CLs are generally classified into three groups (Fig. 2A): Class I isoforms, which are constitutively expressed and primarily associated with lignin biosynthesis in vascular tissues, and Class II isoforms, which are often stress-inducible and closely linked to the production of soluble phenolic compounds such as flavonoids [11-13]. Identifying and characterizing Class II 4CL isoforms is therefore crucial for understanding the regulation of flavonoid accumulation in medicinal plants. In addition to Classes I and II, phylogenetic analyses have identified a Class III 4CL group, which is predominantly found in monocotyledonous plants (monocots) and flowering plants (angiosperms). Similar to Class I 4CLs, Class III members are primarily involved in the biosynthesis of monolignols for lignin deposition and cell wall formation [14].

*Sageretia thea* (*Rhamnaceae*), a shrub widely distributed across East Asia, has long been used in traditional medicine to treat various ailments, including hepatitis, fevers, and skin inflammation [15]. Modern pharmacological studies have confirmed its medicinal value, revealing that it is rich in phenolic acids and flavonoids with potent antioxidant, antidiabetic, and skin-whitening activities [16, 17]. Despite this therapeutic potential, the genetic and biochemical basis of secondary metabolism in *S. thea* remains poorly understood. Given the central role of 4CL in phenylpropanoid metabolism, cloning and characterizing this enzyme in *S. thea* provides an opportunity to elucidate its function, expression patterns, and contribution to plant development and stress responses. Such insights may ultimately facilitate strategies to improve the yield of bioactive compounds from this species.

In this study, we isolated a novel 4CL gene (*St4CL1*) from the transcriptome of *S. thea* and characterized its function through heterologous expression in *Escherichia coli*. Additionally, we investigated its biochemical properties, including optimal catalytic conditions and substrate specificity, to clarify its metabolic role in flavonoid biosynthesis. Furthermore, homology modeling and molecular docking were employed to provide structural insights into substrate binding. This work represents the first report of molecular cloning and biochemical characterization of a functional 4CL from *S. thea*, establishing a foundation for metabolic engineering of phenylpropanoids in this medicinal plant and addressing a critical knowledge gap in its secondary metabolism.

## Materials and Methods

### Chemicals and Materials

All chemicals used in this study were of analytical grade unless otherwise specified. Hydroxycinnamic acid derivatives—including *p*-coumaric acid, caffeic acid, ferulic acid, cinnamic acid, and sinapic acid—were purchased from Sigma-Aldrich (USA) and used both as substrates for enzyme assays and as standards for high-performance liquid chromatography (HPLC). Methanol used to terminate enzymatic reactions was purchased from Thermo Fisher Scientific (USA). HPLC-grade acetonitrile and formic acid, used as mobile phase components, were purchased from Thermo Fisher Scientific and Sigma-Aldrich, respectively. KOD One PCR Master Mix was obtained from Toyobo (Japan), and Gibson Assembly Master Mix was purchased from New England Biolabs (USA) for DNA assembly. The expression vector pET-28a(+) and *E. coli* BL21(DE3) competent cells were purchased from Enzynomics (Republic of Korea) for recombinant protein expression.

### Identification and Expression Profiling of St4CL Genes

To identify the 4-coumarate:CoA ligase (4CL) gene family in *S. thea*, a genome-wide survey was performed using the chromosomal-level genome assembly (https://doi.org/10.6084/m9.figshare.25877698) [18]. Potential 4CL candidates were identified via BLASTp search against the *S. thea* proteome using *Arabidopsis thaliana* 4CL protein sequences as queries, followed by orthology validation using OrthoFinder [19]. To determine the functional importance of the identified paralogs, their transcript abundance in *S. thea* leaves was quantified using RNA-seq data. Raw reads were mapped to the reference genome using STAR v2.7.11 [20], and transcripts were assembled and quantified as Fragments Per Kilobase of transcript per Million mapped reads (FPKM) using Cufflinks v2.2.1 [21].

### Multiple Sequence Alignment and Phylogenetic Analysis

The evolutionary relationship of St4CLs was analyzed alongside 4CL protein sequences from various plant species, with accession numbers listed in Table S1. Multiple sequence alignment was performed using MAFFT v7.505 [22] with the –localpair and --maxiterate 1000 options. The alignment was subsequently trimmed using trimAl v1.5 [23] with a gap threshold of 0.5 to remove poorly aligned regions. A maximum-likelihood phylogenetic tree was constructed using IQ-TREE v2.3.5 [24] with the best-fit amino acid substitution model (MFP) and 1,000 ultrafast bootstrap replicates. The resulting phylogenetic tree was visualized and annotated using FigTree v1.4.4 (http://tree.bio.ed.ac.uk/software/figtree/) to classify the St4CL isoforms.

### Cloning, Culture Conditions, and Sequence Analysis

All bacterial strains, plasmids, and primers used in this study are listed in Table 1 and Table S2. The St4CL gene was cloned into the pET-28a(+) vector containing a kanamycin resistance marker using Gibson Assembly (New England Biolabs). *Escherichia coli* DH5α was used for plasmid propagation and verification of cloning, while *E. coli* BL21 (DE3) was employed for recombinant protein expression. Transformants were cultured in Luria–Bertani (LB) medium (10 g/L tryptone, 5 g/L yeast extract, and 10 g/L NaCl) supplemented with 50 μg/mL kanamycin at 37°C with shaking at 250 rpm.

The amino acid sequence of St4CL1 (Table S4) was extracted from transcriptome data provided by the Honam National Institute of Biological Resources (HNIBR; Republic of Korea). The coding region of the *St4CL1* gene (Table S3) was codon-optimized for heterologous expression in *E. coli* and chemically synthesized by Cosmogenetech (Republic of Korea). Gene-specific primers were synthesized by Enzynomics (Republic of Korea), and cloned sequences were verified by DNA sequencing (Macrogen, Republic of Korea). Multiple sequence alignment [25] was performed using Clustal Omega (EMBL-EBI, UK; accessed in November 2025). Conserved catalytic motifs and sequence similarities were identified, and alignments were visualized and annotated using Jalview (version 2.11.5.0; University of Dundee, UK). This analysis supported functional classification and provided a structural basis for subsequent homology modeling.

### Heterologous Expression and Purification

Recombinant St4CL1 was expressed in *E. coli* BL21(DE3) cells harboring the pET-28a(+) vector. Cultures were induced with 0.1 mM isopropyl β-D-1-thiogalactopyranoside (IPTG) when the optical density at 600 nm (OD_600_) reached 0.6–0.8, measured using a dual-beam UV–visible spectrophotometer (UV-1900i Plus, Shimadzu, Japan). Cells were harvested by centrifugation at 4,000 rpm for 30 min at 4°C, resuspended in lysis buffer (50 mM Tris-HCl, pH 7.5, 200 mM NaCl, 1 mM phenylmethanesulfonyl fluoride [PMSF]) at 10% of the original culture volume, and disrupted on ice using a microtip probe sonicator (Vibra-Cell; Sonics & Materials, Inc., USA) with a 15 s ON/15 s OFF cycle at 25% amplitude for a total effective time of 1 min.

The soluble fraction was purified using Ni^2+^-charged Nuvia IMAC resin (Bio-Rad Lab. USA) packed in an Econo-Column (2.5 × 30 cm; Bio-Rad Laboratories). The column was washed with buffer (50 mM Tris-HCl, pH 7.5, 200 mM NaCl, and 25 mM imidazole) for 10 column volumes (CV), and His-tagged proteins were eluted with a buffer containing 50 mM Tris-HCl, pH 7.5, 200 mM NaCl, and 250 mM imidazole for 2 CV. Protein concentration was determined using the Bradford assay with bovine serum albumin as a standard, and purity was assessed using Coomassie brilliant blue staining following 12% SDS-PAGE.

### Enzyme Activity Assay and Product Analysis

The catalytic activity of St4CL1 was measured using *p*-coumaric acid as a substrate. The standard reaction mixture contained 50 mM Tris-HCl (pH 7.5), 1 mM *p*-coumaric acid, 1 mM coenzyme A (CoA), 5 mM MgCl_2_, and 5 mM ATP. The reaction was initiated by adding purified enzyme and incubating at 30°C for 1 h. An equal volume of ice-cold methanol was then added to terminate the reaction.

The biochemical properties of St4CL1 were investigated by systematically assessing factors influencing catalytic efficiency. First, the effect of enzyme concentration was examined in the range of 0.025–1 mg/mL to determine the optimal protein amount required for analysis. The optimal conditions for St4CL1 activity were further evaluated at different temperatures (20–50°C) and pH values (6.0–10.0). Additionally, the effects of various metal ions (Mn^2+^, Zn^2+^, Cu^2+^, Ni^2+^, Co^2+^, Mg^2+^, Ca^2+^, and Fe^2+^) on enzyme activity were tested at a concentration of 5 mM. Dose-dependent responses to selected ions were subsequently assessed over a concentration range of 0–20 mM.

Reaction products were analyzed using an HPLC system equipped with a UV detector. Samples were separated on a C18 column (4.6 × 250 mm, 3 μm; CAPCELL PAK, Japan) with a mobile phase consisting of 0.1% (v/v) formic acid in water (solvent A) and acetonitrile (solvent B). Gradient elution was performed at a flow rate of 1.0 mL/min under the following profile: 0–5 min, 10% B; 5–10 min, 10%–40% B; 10–20 min, 40%–20% B; and 20–25 min, 20%–10% B. Absorbance was monitored at 340 nm. Because commercial *p*-coumaroyl-CoA is not readily available, enzyme activity was quantified by measuring substrate consumption, comparing peak areas of *p*-coumaric acid before and after the reaction. In addition, time-course HPLC analyses were performed to monitor substrate depletion and the appearance of a putative product peak. Reaction mixtures containing active enzyme were sampled at different incubation times (20, 30, and 60 min) and compared with no-enzyme and heat-denatured enzyme controls.

To determine whether St4CL1 belongs to Class I or Class II 4CL, substrate specificity analysis was performed using representative hydroxycinnamic acid derivatives. Each substrate was tested at a final concentration of 1 mM under optimized conditions. Enzyme activity was measured at 280 nm for cinnamic acid and at 340 nm for all other substrates. Standard curves were generated for each substrate at their respective detection wavelengths (Supplementary Fig. S2). Peak areas were converted to molar concentrations using substrate-specific calibration curves, and specific activity was calculated based on substrate consumption normalized to reaction time and protein amount (μmol·min^−1^·mg^-1^). Relative activity was calculated from specific activity values, with the highest activity set to 100%. All measurements were corrected against no-enzyme and heat-denatured enzyme controls.

### Homology Modeling and Molecular Docking

To elucidate the structural basis of St4CL1 catalysis, a three-dimensional (3D) structural model was constructed using Discovery Studio Client 2026 (Dassault Systèmes BIOVIA, USA). The amino acid sequence of St4CL1 was used as a query to identify homologous templates in the Protein Data Bank (PDB). The crystal structure of *Nicotiana tabacum* 4CL in complex with ATP and Mg^2+^ (PDB ID: 5BSM), representing the catalytically relevant pre-adenylation state, was selected as the template [26]. Twenty models were generated using the Build Homology Models protocol (MODELLER algorithm) and evaluated by the Discrete Optimized Protein Energy (DOPE) score and Probability Density Function (PDF) total energy. The final model was selected based on overall model quality, considering both DOPE scores and PDF total energy values (Table S5). Model quality was validated using the SAVES server, including PROCHECK (Ramachandran analysis) and ERRAT (overall quality factor). The ATP and Mg^2+^ coordinates were manually transferred from the 5BSM template onto the St4CL1 model by structural superposition using PyMOL Molecular Graphics System version 3.1.0 (Schrödinger, LLC). The resulting protein–cofactor complex was prepared using the Prepare Protein protocol and energy-minimized using the CHARMm force field prior to docking.

Molecular docking simulations were performed to investigate the binding modes of St4CL1 toward five hydroxycinnamic acid substrates (*p*-coumaric acid, caffeic acid, ferulic acid, cinnamic acid, and sinapic acid). Potential ligand-binding cavities within the ATP/Mg^2+^-bound St4CL1 model were identified using the Binding Site Analysis protocol. Predicted cavities were evaluated based on their spatial overlap with the ATP/Mg^2+^ binding region derived from the template structure (PDB ID: 5BSM), the presence of conserved catalytic residues, and their location within the characteristic inter-domain cleft of plant 4-coumarate:CoA ligases.

The 3D structures of the ligands were retrieved from the PubChem database and prepared using the Prepare Ligands protocol, followed by energy minimization. Molecular docking was performed using the CDOCKER protocol, a CHARMm-based molecular dynamics simulated-annealing algorithm that generates and refines ligand conformations within the defined binding pocket. Ten poses were generated for each ligand and ranked according to CDOCKER energy and CDOCKER interaction energy. Binding energies were subsequently estimated using the Calculate Binding Energies protocol. Protein–ligand interactions and interacting residues were analyzed using Discovery Studio, and structural visualization was performed using PyMOL.

## Results and Discussion

### Molecular Identification and Phylogenetic Analysis of St4CLs

Through a genome-wide survey of the *S. thea* genome, we identified three putative 4CL genes, designated as *St4CL1* (*ST_chr02.2084*), *St4CL2* (*ST_chr12.583*), and *St4CL3* (*ST_chr09.1195*). To clarify their functional roles, we analyzed their transcript abundance and phylogenetic relationships. Among the three paralogs, *St4CL1* exhibited the highest expression level in leaves (FPKM 120.4), significantly outperforming *St4CL2* (FPKM 52.5) and *St4CL3* (FPKM 0.2). This predominant expression suggests that *St4CL1* is the putative major isoform in leaf secondary metabolism. Although *St4CL2* also belongs to Class II and shows a significant expression level (FPKM 52.5), *St4CL1* was prioritized for further biochemical characterization in this study due to its markedly higher transcript abundance (FPKM 120.4), suggesting it may play a more predominant role in the flavonoid biosynthetic pathway of *S. thea*. However, high gene expression levels of *St4CL1* alone are insufficient to conclude its key role *in vivo*, as actual protein activity is also influenced by variables such as post-translational modifications. To confirm its biological role, additional functional verification is required, such as gene knockout, RNA interference, and comparative analyses with other isoforms.

Phylogenetic analysis further revealed that the *S. thea* 4CL family is divided into two distinct clades (Fig. 2). St4CL1 and St4CL2 clustered within the Class II 4CL group, which is typically associated with the biosynthesis of flavonoids and other soluble phenolic compounds. In contrast, St4CL3 was classified into the Class I group, which generally functions in lignin biosynthesis and vascular development [27]. Based on its predominant transcript abundance and phylogenetic position as a Class II isoform, St4CL1 was selected as the primary target for further biochemical characterization.

Multiple sequence alignments with representative plant 4CLs confirmed that St4CL1 possesses highly conserved motifs, including the AMP-binding motif (Box I: SSGTTGLPKGV) and the catalysis-related motif (Box II: GEICIRG) (Fig. 2) [28]. The clustering of St4CL1 with flavonoid-associated Class II 4CLs, combined with its high transcript abundance, identifies it as a promising enzymatic target for metabolic engineering to enhance flavonoid production in *S. thea*.

Furthermore, the identification of St4CL2 as another Class II member with substantial expression suggests potential functional redundancy or specialization among 4CL isoforms in *S. thea*. While St4CL1 appears to be the primary isoform involved in flavonoid-associated metabolic flux based on its high FPKM value, St4CL2 might contribute to specific physiological responses or be expressed in a tissue-specific manner that was not fully captured in the current transcriptomic data. Therefore, comparative studies between St4CL1 and St4CL2, particularly regarding their substrate preferences and spatio-temporal expression patterns, will be required to elucidate their synergistic or distinct roles in plant secondary metabolism.

### Heterologous Expression and Purification of Recombinant St4CL1

**Fig. 3:**
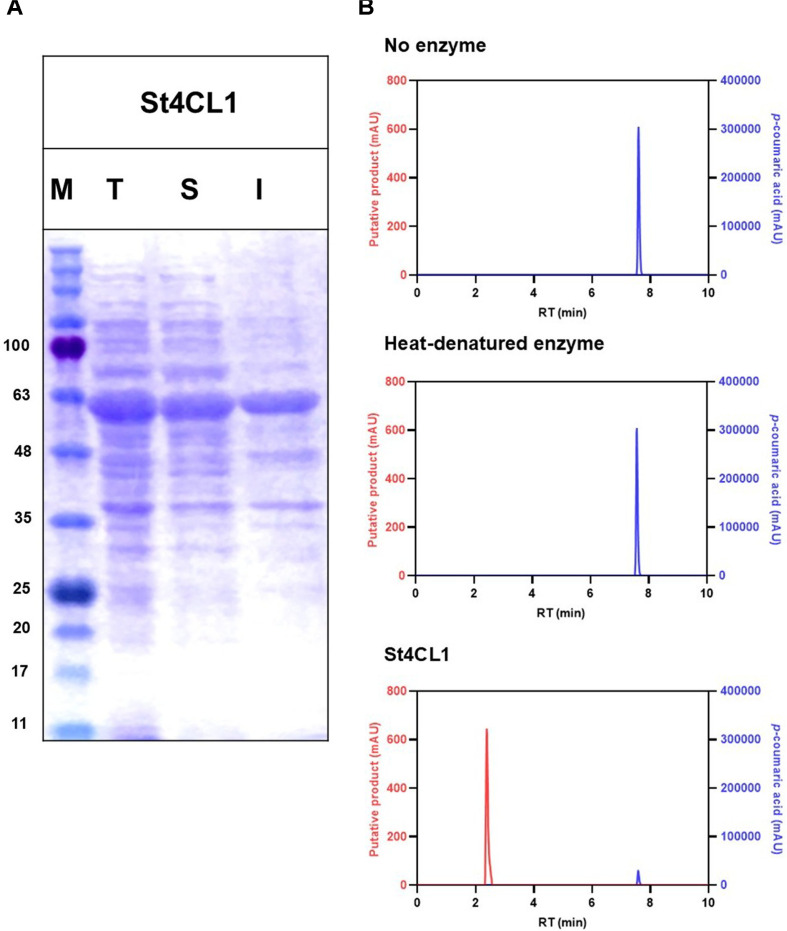
Expression and enzymatic activity of St4CL1. (**A**) SDS-PAGE analysis confirming protein expression of St4CL1 in *E. coli*. (M: Marker, T: Total protein, S: Soluble fraction, I: Insoluble fraction). (**B**) HPLC chromatograms showing putative product formation and *p*-coumaric acid consumption in reactions containing no enzyme, heat-denatured enzyme, or active St4CL1. Red traces represent the putative product, and blue traces represent *p*-coumaric acid.

High-level expression of soluble recombinant proteins is often a bottleneck in characterizing plant enzymes. In this study, the *St4CL1* gene was cloned into the pET-28a(+) expression vector and transformed into *E. coli* BL21(DE3). SDS-PAGE analysis confirmed the successful expression of the fusion protein, revealing a distinct band at ~59.5 kDa, consistent with the predicted molecular weight (Fig. 3A). Unlike many plant enzymes that form inclusion bodies, a substantial fraction of St4CL1 was recovered in the soluble fraction, indicating proper folding in the bacterial host.

The catalytic function of purified St4CL1 was verified through *in vitro* activity assays using *p*-coumaric acid as a substrate. As commercial *p*-coumaroyl-CoA standards were not readily available, enzymatic activity was evaluated by monitoring both substrate depletion and the time-dependent appearance of a putative product peak using HPLC analysis [29]. No significant substrate depletion or product peak formation was observed in the no-enzyme and heat-denatured enzyme controls (Fig. 3B). In contrast, reactions containing active St4CL1 resulted in a marked decrease in the *p*-coumaric acid peak and the appearance of a new peak at a retention time of approximately 2.3 min (Fig. 3B). Although the identity of this putative product peak could not be definitively confirmed due to the absence of an authentic *p*-coumaroyl-CoA standard, it was absent in both negative controls and exhibited a time-dependent increase over the 60-min reaction period (Fig. S1). These characteristics are consistent with an enzyme-dependent product, collectively supporting the role of St4CL1 as a functional 4-coumarate:CoA ligase involved in the phenylpropanoid pathway in *S. thea*.

It should be noted, however, that while these *in vitro* data characterize the fundamental enzymatic potential of St4CL1, it is essential to interpret these properties within the context of the *E. coli* heterologous expression system. Although this system is a robust and widely used platform for characterizing plant 4CLs, it lacks the complex machinery required for eukaryotic post-translational modifications (PTMs), such as glycosylation or phosphorylation. In the native plant environment, such modifications can significantly modulate enzyme stability, catalytic efficiency, or sub-cellular localization. Therefore, the biochemical parameters reported here reflect the intrinsic catalytic capacity of the St4CL1 polypeptide sequence, which may lack the regulatory fine-tuning provided by PTMs *in vivo*. Future studies utilizing plant-based expression systems or in planta functional characterization—such as gene knockout or RNA interference—will be pivotal to fully deciphering the impact of PTMs on the physiological role of St4CL1.

### Biochemical Characterization and Catalytic Properties

**Fig. 4:**
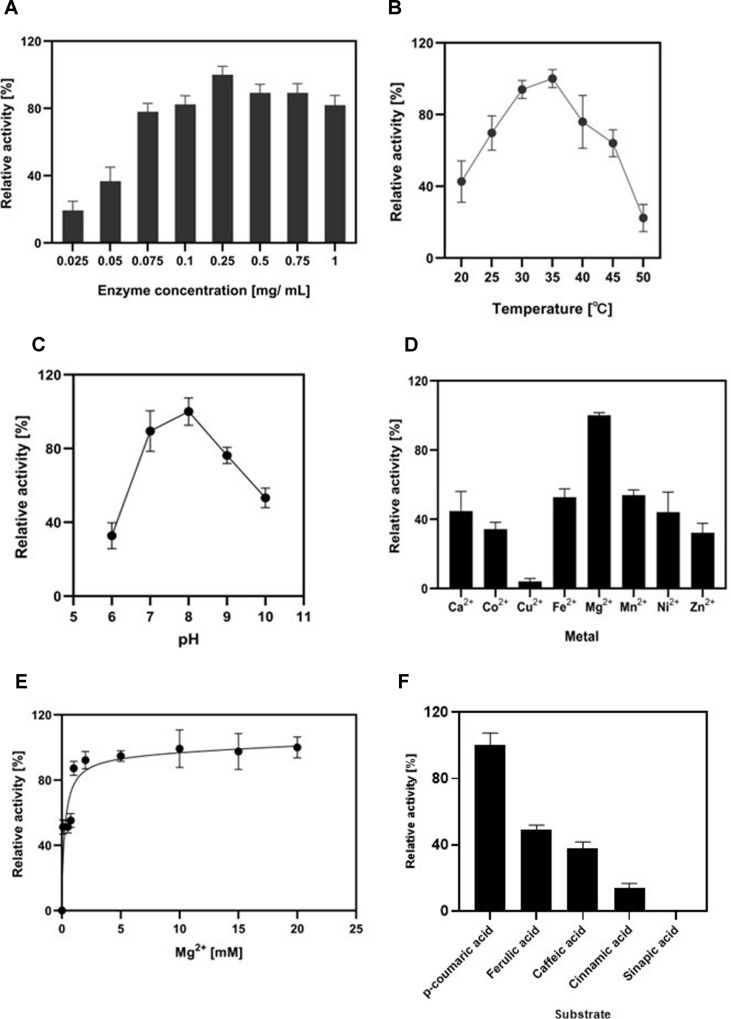
Optimization of recombinant St4CL1 reaction conditions. (**A**) Effect of enzyme concentration (0.025–1 mg/mL) on St4CL1 activity. (**B**) Temperature profile of St4CL1 activity ranging from 20 to 50°C. (**C**) Effect of pH (6.0–10.0) on St4CL1 activity. (**D**) Effect of divalent metal cations (5 mM each) on enzymatic activity. (**E**) Effect of Mg^2+^ concentration (0–20 mM) on St4CL1 activity. All reactions were performed using *p*-coumaric acid as the substrate. (**F**) Substrate specificity of St4CL1 toward different hydroxycinnamic acids (1 mM each), including *p*-coumaric, ferulic, caffeic, cinnamic, and sinapic acids. Data are expressed as mean ± SEM from three independent experiments (*n* = 3).

The effect of enzyme concentration on substrate consumption was first examined to determine the linear range of the reaction. St4CL1 exhibited concentration-dependent activity, with the reaction rate reaching a plateau at 0.25 mg/mL (Fig. 4A). Accordingly, 0.25 mg/mL was selected as the optimal enzyme loading for standardized assays.

Environmental factors strongly influence catalytic efficiency. Therefore, the optimal temperature and pH for St4CL1 were determined. St4CL1 displayed maximal activity at 35°C (Fig. 4B). The enzyme remained stable between 30°C and 40°C, but activity declined sharply above 45°C, likely due to thermal denaturation of the tertiary structure. This thermal profile resembles that of other mesophilic plant 4CLs, reflecting adaptation to moderate growth conditions. For pH dependence, St4CL1 exhibited a bell-shaped profile with optimal activity at pH 7.0–8.0 (Fig. 4C). Activity decreased significantly below pH 6.0 and above pH 9.0, consistent with the altered ionization states of catalytic residues or substrate dissociation. This pH optimum aligns with the cytosolic pH of plant cells, supporting the physiological role of St4CL1 in cytoplasmic flavonoid biosynthesis [30].

As an ATP-dependent ligase belonging to the adenylate-forming enzyme family, 4CL requires divalent metal ions for catalytic activity. In this study, St4CL1 activity showed a strong preference for metal cofactors, particularly Mg^2+^ (Fig. 4D). Among the cations tested, Mg^2+^ was the most effective activator, whereas ions such as Cu^2+^ showed markedly lower efficacy. Concentration-dependent assays revealed that St4CL1 activity increased linearly with Mg^2+^ concentration up to 5 mM (Fig. 4E). Mechanistically, Mg^2+^ coordinates with the phosphate groups of ATP to form the Mg-ATP^2-^ complex, which serves as the true substrate, facilitating nucleophilic attack during adenylate formation [31].

Substrate specificity was assessed using hydroxycinnamic acid derivatives (Fig. 4F). Relative activity was determined from specific activity values (μmol·min^-1^·mg^-1^) calculated using substrate-specific calibration curves (Supplementary Fig. S2). *p*-Coumaric acid supported the highest catalytic activity (100%) , whereas ferulic acid and caffeic acid showed intermediate activity (49.1% and 38.0%, respectively). Cinnamic acid exhibited low activity (14.1%), while no detectable activity was observed toward sinapic acid. This substrate preference pattern is characteristic of flavonoid-associated Class II 4CLs and distinguishes St4CL1 from lignin-associated Class I 4CLs, which generally exhibit broader substrate tolerance. Collectively, these results provide strong biochemical evidence that St4CL1 functions as a Class II 4CL involved in flavonoid biosynthesis rather than lignin formation [7, 32].

### Homology Modeling and Structural Analysis of St4CL1

To elucidate the 3D structural features of St4CL1, a homology model was constructed using the crystal structure of *N. tabacum* 4CL (Nt4CL; PDB ID: 5BSM) as a template [26]. Nt4CL was selected because it exhibits high sequence identity (79.9%) and similarity (90.1%) with St4CL1, and the 5BSM structure is solved in complex with ATP and Mg^2+^, capturing the enzyme in the catalytically relevant pre-adenylation state. Among the 20 generated models, model M0014 was selected based on its overall model quality, with favorable DOPE scores and PDF total energy values (Table S5). Ramachandran plot analysis confirmed that 93.9% of residues occupy favored regions with no disallowed outliers, and the model returned an ERRAT overall quality factor of 82.0%, indicating good stereochemical quality (Fig. S3). Superposition on the template yielded a Cα RMSD of 0.12 Å, confirming that the model faithfully reproduces the template fold.

**Fig. 5:**
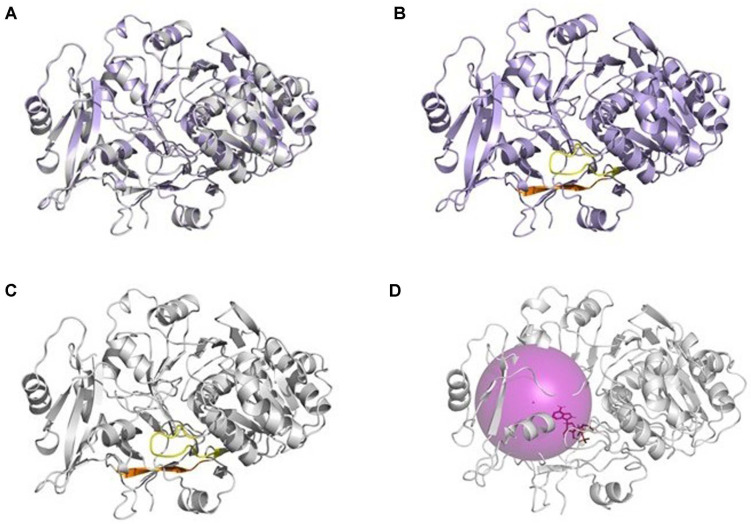
Structural comparison between the predicted St4CL1 model and the Nt4CL crystal structure. (**A**) Structural superimposition of the predicted St4CL1 model (purple) and Nt4CL (PDB ID: 5BSM, gray) showing high overall similarity. (**B**) Ribbon representation of the predicted St4CL1 structure. (**C**) Ribbon representation of the Nt4CL crystal structure. Conserved Box I and Box II motifs are highlighted in yellow and orange, respectively, demonstrating their spatial conservation between St4CL1 and Nt4CL. (**D**) Predicted principal substrate-binding cavity (Site 1; magenta sphere) of the St4CL1 model, located within the inter-domain cleft and overlapping the ATP/Mg^2+^ region (shown as sticks) transferred from the Nt4CL template.

The predicted St4CL1 structure adopts the canonical fold of the adenylate-forming enzyme superfamily, comprising a large N-terminal domain and a smaller C-terminal domain connected by a flexible hinge region [33]. This bilobed organization, together with the active-site architecture, was conserved relative to Nt4CL (Fig. 5A). The AMP-binding motif (Box I; SSGTTGLPKGV) and catalytic motif (Box II; GEICIRG) are spatially conserved between St4CL1 and Nt4CL (Figs. 5B and 5C), with Box I positioning ATP phosphate interactions and Box II located at the N–C terminal interface. The conservation of these functional motifs, together with the structural validation above, corroborates the biochemical identification of St4CL1 as a functional 4CL.

ATP and Mg^2+^ coordinates were transferred from the Nt4CL template onto the St4CL1 model, and Binding Site Analysis identified Site 1 as the principal cavity, located within the inter-domain cleft between Box I and Box II and overlapping the ATP/Mg^2+^ region (Fig. 5D). Site 1 was therefore selected for docking of the five hydroxycinnamic acid substrates.

**Fig. 6:**
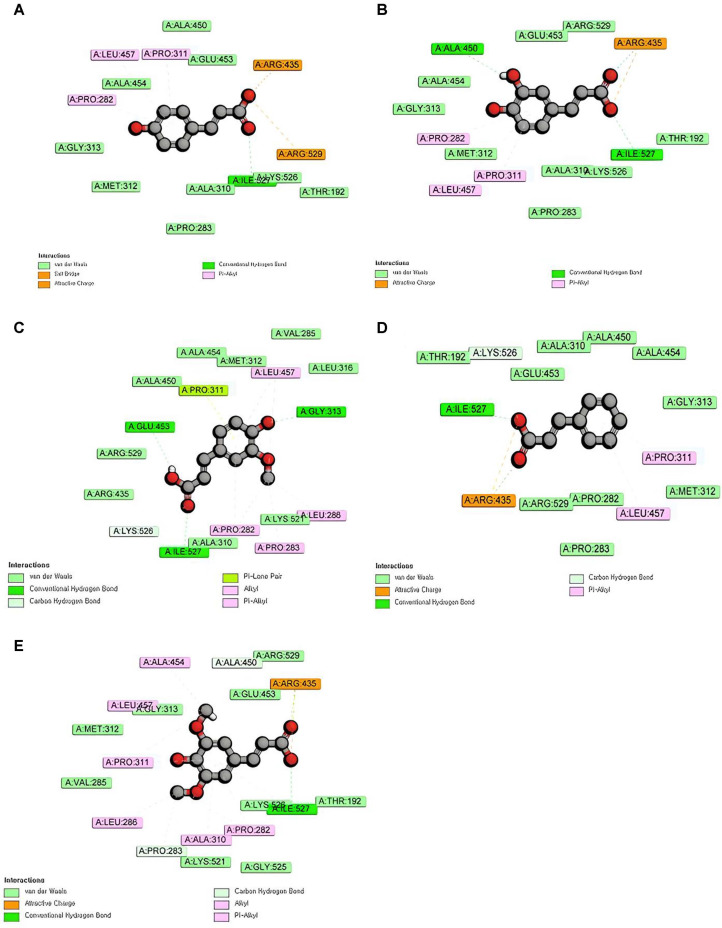
Two-dimensional (2D) interaction diagrams of hydroxycinnamic acid substrates docked into Site 1 of St4CL1. (**A**) *p*-Coumaric acid, showing a salt-bridge contact between the carboxylate and Arg435. (**B**) Caffeic acid. (**C**) Ferulic acid. (**D**) Cinnamic acid. (**E**) Sinapic acid. Residues lining the binding pocket are labeled, and interaction types are color-coded as indicated in the legend of each panel.

All five substrates docked into Site 1 with the carboxylate contacting Arg435 and Ile527 and the aromatic ring buried in a hydrophobic sub-pocket lined by Pro282, Pro283, Ala310, Pro311, Met312, Gly313, Ala454, and Leu457 (Fig. 6A–6E; Table 2). The carboxylate–Arg contacts of *p*-coumaric acid were classified as a salt bridge in the Discovery Studio interaction analysis, whereas the equivalent contacts in the other four substrates were assigned as attractive-charge or van der Waals interactions (Fig. 6A–6E). The bulkier ferulic and sinapic acids additionally contacted Val285, with sinapic acid further contacting Leu286, consistent with the larger volume occupied by their methoxylated rings (Fig. 6C and 6E). By −CDOCKER interaction energy and binding free energy, sinapic acid ranked highest (45.03 and −100.77 kcal/mol), followed by caffeic, ferulic, *p*-coumaric, and cinnamic acid (Table 2).

This docking ranking did not match the measured catalytic activities, in which *p*-coumaric acid was the preferred substrate and sinapic acid was inactive (Fig. 4F). This dissociation between docking energetics and catalytic activity is consistent with previous CDOCKER benchmarks reporting that docking energies optimize complex stability rather than the geometric criteria required for catalysis [34] ; the additional Val285/Leu286 contacts of the bulkier ferulic and sinapic acids enlarge the contact surface and increase the apparent docking score without indicating catalytic competence. The activity ranking is better understood in terms of binding mode together with the substrate-discriminating residues defined for Nt4CL2. *p*-Coumaric acid is the only substrate whose carboxylate–Arg contact was classified as a salt bridge, while the inactivity of sinapic acid is consistent with the steric incompatibility of 3,5-disubstituted substrates with a catalytically productive pose, owing to the Gly339–Leu342 region (containing Val341) defined in Nt4CL2, where mono-meta substituents like the 3-methoxy of ferulate are instead accommodated in the larger-volume region opposite [7, 26]. These regions are spatially conserved in St4CL1 and account for the extremes of the activity ranking — the preference for *p*-coumaric acid and the inactivity of sinapic acid — despite the latter's tight predicted binding. These observations indicate that substrate selectivity in St4CL1 is governed by binding mode rather than by binding affinity, a feature characteristic of Class II 4CLs in flavonoid biosynthesis, consistent with the structural framework established for plant 4CL substrate recognition [7].

## Conclusion

In this study, a novel 4CL gene (*St4CL1*) was identified and functionally characterized from the medicinal plant *S. thea*, providing the first molecular insight into its phenylpropanoid metabolism. By integrating phylogenetic analysis, biochemical characterization, and structural modeling, we demonstrated that St4CL1 functions as a Class II isoform that channels carbon flux toward the biosynthesis of bioactive flavonoids rather than lignin. Biochemical assays established that recombinant St4CL1 operates optimally under physiological conditions (pH 7.0–8.0 and 35°C) with a strong preference for Mg^2+^, while molecular docking simulations indicated that substrate selectivity is governed by binding mode rather than by binding affinity, with *p*-coumaric acid uniquely forming a salt-bridge interaction at the carboxylate-binding site that distinguishes it from the other hydroxycinnamic acid substrates tested. These findings fill a critical gap in understanding the secondary metabolism of *S. thea* and provide a valuable genetic resource for metabolic engineering and synthetic biology. Future studies focusing on the in vivo regulation of the *St4CL1* gene and the interactions of the St4CL1 protein with downstream enzymes will further enable the sustainable production of plant-derived therapeutics.

## Supplemental Materials

Supplementary data for this paper are available on-line only at http://jmb.or.kr.



## Figures and Tables

**Table 1:** Bacterial strains and plasmids used in this study.

Name	Description	Reference
Strains		
DH5α	*E. coli*, *F− Φ80lacZΔM15 Δ(lacZYA-argF)U169 deoR recA1 endA1 hsdR17(rk−, mk+) phoA supE44 thi-1 gyrA96 relA1*	[35]
BL21(DE3)	*E. coli*, *F− dcm ompT hsdS(rB−, mB−) gal (DE3)*	[35]
BL21-St4CL	*E. coli* BL21(DE3) harboring pET28-St4CL	In this study
Plasmids		
pET28a(+)	P_T7_, pBR322 ori, Km^R^	[35]
pET28-St4CL	pET28a(+) containing P_T7_-St4CL-His	In this study

**Table 2:** Molecular docking energy parameters of St4CL1 with different phenylpropanoid substrates.

Substrate	-CDOCKER Energy (kcal/mol)	-CDOCKER Interaction Energy (kcal/mol)	Binding Free Energy (kcal/mol)
*p*-coumaric acid	30.7	34.2952	-70.0785
Caffeic acid	35.6928	37.16	-74.6088
Ferulic acid	29.4253	34.6315	-53.8085
Cinnamic acid	27.2478	31.2265	-69.1089
Sinapic acid	34.1716	45.0313	-100.7739
