# Supplementary material for: Molecular Cloning, Biochemical Characterization, and Structural Insights into a Flavonoid-Associated Class II 4-Coumarate:CoA Ligase from Sageretia thea
Source: J Microbiol Biotechnol. 2026 Jun 29;36:e2604014. doi: 10.4014/jmb.2604.04014 (PMC13341628; doi:10.4014/jmb.2604.04014)

J. Microbiol. Biotechnol. 2026. 36: e2604014

https://doi.org/10.4014/jmb.2604.04014

Molecular Cloning, Biochemical Characterization, and Structural Insights into a Flavonoid-Associated Class II 4-Coumarate:CoA Ligase from *Sageretia thea*

Yo-Heun Kim[1†], Shin Ae Lee[2†], Ji-Eun Kim[3], Minseok Cha[3], Jihoon Jo[4], Kyung Jun Lee[5*], and Soo-Jung Kim[1,3*]

[1] Department of Integrative Food, Bioscience and Biotechnology, Chonnam National University, Gwangju 61186, Republic of Korea

[2] Division of Biodiversity Conservation, Honam National Institute of Biological Resources, Jeollanam-do 58672, Republic of Korea

[3] Research Center for Biological Cybernetics, Chonnam National University, Gwangju 61186, Republic of Korea

[4] Division of Genetic Diverisity, Honam National Institute of Biological Resources, Jeollanam-do 58672, Republic of Korea

[5] Division of Bioresource Integration Research, Honam National Institute of Biological Resources, Jeollanam-do 58672, Republic of Korea

[†]These authors contributed equally to this work.

* Corresponding authors:

Kyung Jun Lee

E-mail address: lkj5214@hnibr.re.kr;

Soo-Jung Kim: E-mail address: bioksj@jnu.ac.kr

Table S1. Accession numbers of 4CL proteins used for phylogenetic analysis.

Species	Name	Accession Number / Gene ID
Sageretia thea	St4CL1	ST_chr02.2084*
Sageretia thea	St4CL2	ST_chr12.583*
Sageretia thea	St4CL3	ST_chr09.1195*
Allium cepa	Ac4CL	AAS48417.1
Angelica sinensis	As4CL	AMP18194.1
Arabidopsis thaliana	At4CL1	AAA82888.1
Arabidopsis thaliana	At4CL2	AAD47193.1
Arabidopsis thaliana	At4CL3	AAD47195.1
Agastache rugosa	Ar4CL	AAT02218.1
Camellia sinensis	Cs4CL	ABA40922.1
Glycine max	Gm4CL1	AAL98709.1
Glycine max	Gm4CL2	AAC97600.1
Glycine max	Gm4CL3	AAC97599.1
Glycine max	Gm4CL4	CAC36095.1
Lithospermum erythrorhizon	Le4CL1	BAA08365.1
Lithospermum erythrorhizon	Le4CL2	BAA08366.2
Lolium perenne	Lp4CL1	AAF37732.1
Lolium perenne	Lp4CL2	AAF37733.1
Lolium perenne	Lp4CL3	AAF37734.1
Nicotiana tabacum	Nt4CL1	AAB18637.1
Nicotiana tabacum	Nt4CL2	AAB18638.1
Ocimum basilicum	Ob4CL	AGP02119.1
Oryza sativa	Os4CL	CAA36850.1
Ocimum tenuiflorum	Ot4CL	ADO16242.1
Petroselinum crispum	Pc4CL1	CAA31696.1
Petroselinum crispum	Pc4CL2	CAA31697.1
Prunus avium	Pa4CL1	ADZ54779.1
Populus trichocarpa x Populus deltoides	Pd4CL1	AAC39365.1
Populus trichocarpa x Populus deltoides	Pd4CL2	AAC39366.1
Pinus radiata	Pr4CL	ACF35279.1
Pinus taeda	Pta4CL1	AAA92668.1
Pinus taeda	Pta4CL2	AAA92669.1
Populus tremuloides	Ptr4CL1	AAC24503.1
Populus tremuloides	Ptr4CL2	AAC24504.1
Panicum virgatum	Pv4CL	ACD02135.1
Panicum virgatum	Pv4CL2	ADZ96250.1
Rubus idaeus	Ri4CL1	AAF91310.1
Rubus idaeus	Ri4CL2	AAF91309.1
Rubus idaeus	Ri4CL3	AAF91308.1
Sorbus aucuparia	Sa4CL3	ADE96997.1
Scutellaria baicalensis	Sb4CL	BAD90937.1
Solanum tuberosum	Stu4CL1	AAA33842.1
Solanum tuberosum	Stu4CL1a	AAD40664.1
Vanilla planifolia	Vp4CL	O24540.1
Zea mays	Zm4CL	AAS67644.1

*Gene IDs from the chromosomal-level genome assembly of *Sageretia thea* (Jo et al., 2024; https://doi.org/10.6084/m9.figshare.25877698) were used. All other sequences were retrieved from the NCBI GenBank database.

Table S2. Oligonucleotide primers used in this study

Primer	Sequence (5′–3′)	Purpose
pET28a_Vector_F	GGTAGCGGTCACCACCACCACCACCACTG	Vector amplification
pET28a_Vector_R	GGTATATCTCCTTCTTAAAGTTAAACAAAA	
pET28a_St4CL_F	TAACTTTAAGAAGGAGATATACCATGGAACATAAACAGTATCAGCAAGAA	St4CL gene amplification
pET28a_St4CL_R	TGGTGGTGGTGACCGCTACCGTTGCACTGACCGCTCGCCA	

Table S3. DNA sequence of the St4CL1 gene

Gene	Nucleotide sequence
St4CL1	TGGAACATAAACAGTATCAGCAAGAATTTATTTTTCGCAGCAAACTG<br>CCGGATATTTATATTCCGAACCATCTGCCGCTGCATAGCTATTGCTTT<br>GAAAACATTAGTCAGTTTAAAGATCGCACCTGCCTGATTGATGGCG<br>CGACCGGCGAAACCTATACCTATAGCGATGTGGAACTGACCGCGCG<br>CAAAGTGGCGGCGGGCCTGGATAAACTGGGCATTCGCCAAGGCGA<br>TGTGATTATGCTGTGGCTGCCGAACTGCACGCAGTTTGTGTTTGCG<br>TTTCTGGGCGCGAGCTATATTGGCGCGATGAGCACCACCGCGAAC<br>CCGTTTTATACCCCGGCGGAAGTGGCGAAACAAGCGAGCGCGAGC<br>AAAGCGAAACTGATTATTACCCAAGCGGCGTATGTGGATAAAGTGAA<br>AGATTTTGCGAAACAGAACGATGTGAAAATTATGTGCATTGATAGCC<br>CGCCGGAAGGCCTGCTGCATTTTAGCGAACTGACCCAAGCGGATG<br>AAAGCGAGATTCCGGCGGTGAAAATTAACCCGGATGATGTGGTGG<br>CGCTGCCGTATAGCAGCGGCACCACCGGCCTGCCGAAAGGCGTG<br>ATGCTGACCCATAAAGGCCTGGTGACGAGCGTGGCGCAGCAAGTG<br>GATGGCGAAAACCCGAACCTGTATTTTCATAGCGAAGATGTGATTCT<br>GTGCGTGCTGCCGCTGTTTCATATTTATAGCCTGAACAGCATTCTGC<br>TGTGCGGCCTGCGCGTGGGCGCGGCGATTCTGATTATGCAGAAAT<br>TTGATGCGAACAAACTGTTTGAACTGGTGGAAAAATATAAAGTGACC<br>ATTGCGCCGTTTGTGCCGCCGATTGTGCTGGCGATTGCGAAAAGC<br>CCGGATGTGAACCGCTATGATCTGAGCAGCGTGCGCATGGTGATG<br>AGCGGCGCGGCGCCGATGGGCAAAGAACTGGAAGATGCGCTGCG<br>CGCGAAACTGCCGAGCGCGAAACTGGGCCAAGGCTATGGCATGAC<br>CGAAGCGGGCCCGGTGCTGAGCATGTGCCTGGCGTTTGCGAAAG<br>AACCGTTTGATATTAAAAGCGGCGCGTGCGGCACCGTGGTGCGCA<br>ACGCGGAAATGAAAATTCTGGATCCGGATACCGGCGCGAGCCTGC<br>CGCGCAACCAAGCGGGCGAAATTTGCATTCGCGGCAGTCAGATTAT<br>GAAAGGCTATCTGAACGATCCGGAAGCGACCGAAAACACCATTGAT<br>AAAGATGGCTGGCTGCATACCGGCGATATTGGCTATATTGATGATGA<br>TGATGAACTGTTTATTGTGGATCGCCTGAAAGAACTGATTAAATATAA<br>AGGCTTTCAAGTGGCGCCGGCGGAACTGGAAGCGCTGCTGCTGG<br>CGCATCCGAACATTAGCGATGCGGCGGTGGTGCCGATGAAAGATG<br>AAGCGGCCGGCGAAATTCCGGTGGCGTTTGTGGTGCGCAGCAAC<br>GGCAGCAAAATTACCGAAGATGATATTAAACAGTATATTAGCAAACAA<br>GTGGTGTATTATAAACGCATTGGCCGCGTGTTTTTTACCGATAAAGT<br>GCCGAAAGCGCCGAGCGGCAAAATTCTGCGCAAAGATCTGCGCGC<br>GCGCCTGGCGAGCGGTCAGTGCAACTAA

Table S4. Amino acid sequence of St4CL1

Protein	Amino acid sequence
St4CL1	MEHKQYQQEFIFRSKLPDIYIPNHLPLHSYCFENISQFKDRTCLIDGAT GETYTYSDVELTARKVAAGLDKLGIRQGDVIMLWLPNCTQFVFAFLGA SYIGAMSTTANPFYTPAEVAKQASASKAKLIITQAAYVDKVKDFAKQND VKIMCIDSPPEGLLHFSELTQADESEIPAVKINPDDVVALPYSSGTTGLP KGVMLTHKGLVTSVAQQVDGENPNLYFHSEDVILCVLPLFHIYSLNSILL CGLRVGAAILIMQKFDANKLFELVEKYKVTIAPFVPPIVLAIAKSPDVNR YDLSSVRMVMSGAAPMGKELEDALRAKLPSAKLGQGYGMTEAGPVL SMCLAFAKEPFDIKSGACGTVVRNAEMKILDPDTGASLPRNQAGEICI RGSQIMKGYLNDPEATENTIDKDGWLHTGDIGYIDDDDELFIVDRLKEL IKYKGFQVAPAELEALLLAHPNISDAAVVPMKDEAAGEIPVAFVVRSNG SKITEDDIKQYISKQVVYYKRIGRVFFTDKVPKAPSGKILRKDLRARLAS GQCN

Table S5. DOPE scores of homology models of St4CL1

Model Scores			
**Name**	**PDF Total Energy**	**PDF Physical Energy**	**DOPE Score**
St4CL.M0018	2,419.99	1,485.51	-66,629.20
**St4CL.M0014**	**2,429.47**	**1,476.44**	**-67,112.70**
St4CL.M0003	2,453.63	1,486.61	-66,717
St4CL.M0006	2,465.20	1,497.25	-67,182.10
St4CL.M0011	2,484.70	1,506.08	-66,940
St4CL.M0004	2,504.69	1,498.38	-66,768.70
St4CL.M0019	2,511.62	1,496.47	-66,772.10
St4CL.M0005	2,551.16	1,504.48	-66,942.70
St4CL.M0015	2,567.08	1,501.97	-66,800.80
St4CL.M0010	2,572.97	1,494.26	-66,704.30
St4CL.M0013	2,606.65	1,509.51	-66,732.40
St4CL.M0020	2,614.58	1,508.67	-66,598.80
St4CL.M0008	2,614.84	1,538.76	-66,623.10
St4CL.M0016	2,615.09	1,509.67	-66,674.70
St4CL.M0009	2,626.90	1,505.99	-66,348.70
St4CL.M0001	2,668.10	1,502.51	-66,708.50
St4CL.M0012	2,693.39	1,554.21	-66,736
St4CL.M0017	2,701.17	1,493.11	-66,541.30
St4CL.M0002	2,703.52	1,516.80	-66,610.30
St4CL.M0007	2,791.35	1,516.66	-66,639.60

Fig. S1. Time-course HPLC analysis of St4CL1 catalytic activity.

(A) Chromatogram region showing the appearance and time-dependent increase of a putative product peak (RT ≈ 2.3 min) in reactions containing active St4CL1. **(B)** Corresponding decrease of the p-coumaric acid substrate peak (RT ≈ 7.6 min). Chromatograms are shown for no-enzyme control, heat-denatured enzyme control, and active St4CL1 incubations at 20, 30, and 60 min. No product formation or appreciable substrate depletion was observed in either control. Representative chromatograms from three independent experiments (n = 3) are shown.

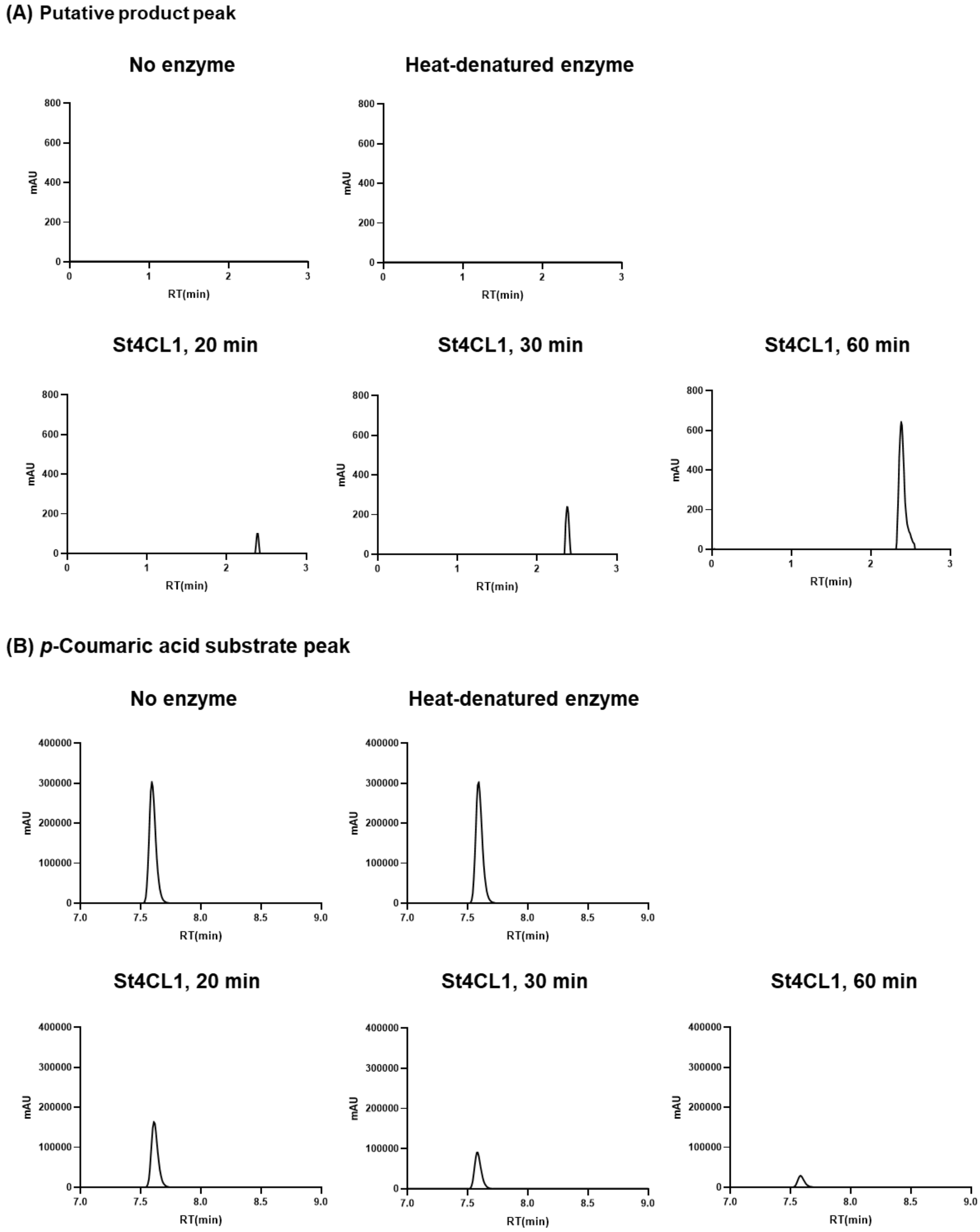

Fig. S2. Calibration curves of hydroxycinnamic acids for quantification. Calibration curves for **(A)** *p*-coumaric acid, **(B)** ferulic acid, **(C)** caffeic acid, **(D)** cinnamic acid, and **(E)** sinapic acid, used to convert HPLC peak areas into molar concentrations for enzyme activity calculations. Concentration ranges and linear regression (R^2) values are indicated for each curve.

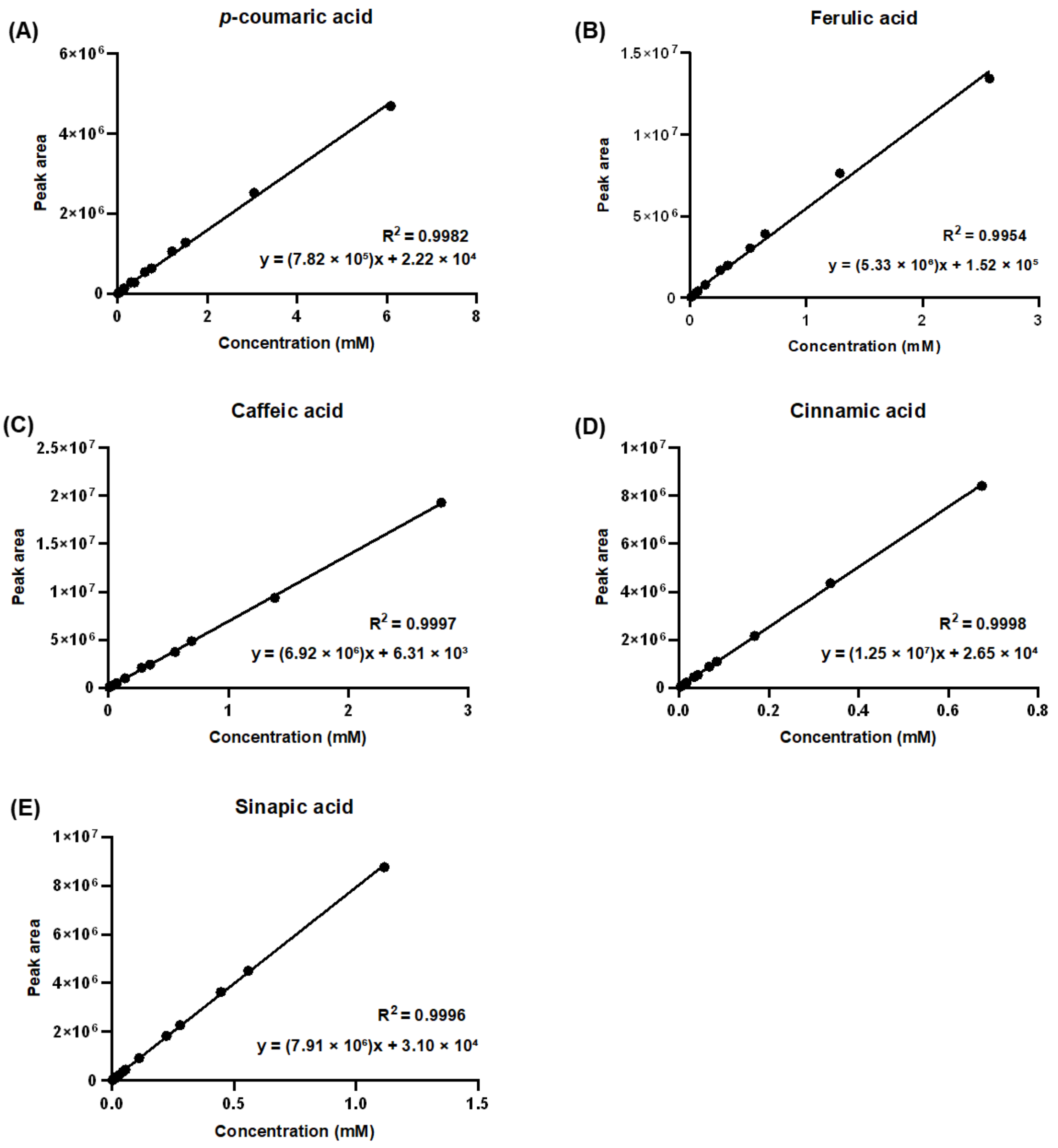

Fig. S3. Ramachandran plot of the St4CL1 protein model.

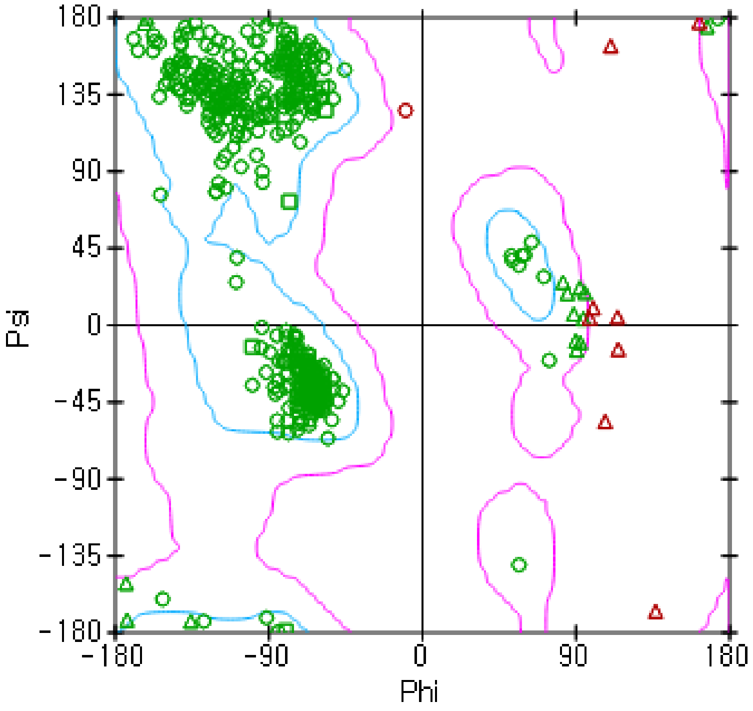

Supplement: Supplementary file 1 [file jmb-36-e2604014-supple.pdf]
